# Pharmacokinetics, Mass Balance, Excretion, and Tissue Distribution of Plasmalogen Precursor PPI-1011

**DOI:** 10.3389/fcell.2022.867138

**Published:** 2022-04-25

**Authors:** Tara Smith, Kaeli J. Knudsen, Shawn A. Ritchie

**Affiliations:** Med-Life Discoveries LP, Saskatoon, SK, Canada

**Keywords:** PPI-1011, plasmalogen augmentation, absorption, excretion, distribution, metabolism

## Abstract

PPI-1011 is a synthetic plasmalogen precursor in development as a treatment for multiple plasmalogen-deficiency disorders. Previous work has demonstrated the ability of PPI-1011 to augment plasmalogens and its effects *in vitro* and *in vivo*, however, the precise uptake and distribution across tissues *in vivo* has not been investigated. The purpose of this study was to evaluate the pharmacokinetics, mass balance, and excretion of [^14^C]PPI-1011 following a single oral administration at 100 mg/kg in Sprague-Dawley rats. Further tissue distribution was examined using quantitative whole-body autoradiography after both single and repeat daily doses at 100 mg/kg/day. Non-compartmental analysis showed that following a single dose, PPI-1011 exhibited peak levels between 6 and 12 h but also a long half-life with mean t_1/2_ of 40 h. Mass balance showed that over 50% of the compound-associated radioactivity was absorbed by the body, while approximately 40% was excreted in the feces, 2.5% in the urine, and 10% in expired air within the first 24 h. Quantitative whole-body autoradiography following a single dose showed uptake to nearly all tissues, with the greatest initial uptake in the intestines, liver, and adipose tissue, which decreased time-dependently throughout 168 h post-dose. Following 15 consecutive daily doses, uptake was significantly higher across the entire body at 24 h compared to single dose and remained high out to 96 h where 75% of the initially-absorbed compound-associated radioactivity was still present. The adipose tissue remained particularly high, suggesting a possible reserve of either plasmalogens or alkyl diacylglycerols that the body can pull from for plasmalogen biosynthesis. Uptake to the brain was also definitively confirmed, proving PPI-1011’s ability to cross the blood-brain barrier. In conclusion, our results suggest that oral administration of PPI-1011 results in high uptake across the body, and that repeated dosing over time represents a viable therapeutic strategy for treating plasmalogen deficiencies.

## Introduction

Plasmalogens are naturally-occurring ethanolamine phospholipids derived in the peroxisome and endoplasmic reticulum that contain a vinyl-ether bond at the *sn*1 position. Plasmalogens play diverse roles within the body, but they primarily function as lipid membrane components modulating cell membrane architecture (Rog and Koivuniemi, 2016), fluidity (Lohner et al., 1984; Hermetter et al., 1989; Lohner et al., 1991) and vesicular fusion (Glaser and Gross, 1994; Glaser and Gross, 1995; Dorninger et al., 2017b). Plasmalogen deficiency is associated with several neurological disorders, including the pediatric rare disease Rhizomelic Chondrodysplasia Punctata (RCDP) (Wanders et al., 1992; Wanders et al., 1994; Braverman et al., 1997), Parkinson’s disease (Dragonas et al., 2009; Fabelo et al., 2011), and Alzheimer’s disease (Han, 2005; Goodenowe et al., 2007). Attention is therefore being given to the potential of plasmalogen replacement as a therapeutic approach for treating these conditions. Although there are some natural dietary sources enriched in plasmalogens including select marine invertebrates such as sea squirts (Yamashita et al., 2017) and scallops (Fujino et al., 2017; Fujino et al., 2020), the concentrations are well below what is anticipated for therapeutic efficacy in humans (Bozelli and Epand, 2021). This leaves the synthetic production of plasmalogens as the only viable and practical approach for clinically-relevant therapeutic intervention.

To address this issue, we have engineered a synthetic plasmalogen precursor, PPI-1011, which has excellent shelf-life stability and is amenable to formulation up to concentrations as high as 500 mg/ml; a concentration high enough to provide the dosing anticipated for clinical efficacy. To our knowledge, PPI-1011 represents the first plasmalogen-augmenting compound undergoing a formal drug development process including preclinical safety-toxicology testing and multi-kilogram GMP manufacturing. PPI-1011 contains an ether-linked, 16:0 fatty alcohol at the *sn*1 position, 22:6 fatty acid (DHA) at the *sn*2 position, and lipoic acid at the *sn*3 position (Wood et al., 2011d). The lipoic acid is a critical component that stabilizes the molecule for long-term storage and prevents migration of the DHA fatty acid from *sn*2 to *sn*3 of the glycerol backbone (Wood et al., 2011a). Endogenously, introduction of the ether bond in the peroxisome is the critical step in plasmalogen biosynthesis, which is catalyzed by alkylglycerone phosphate synthase (AGPS) following the acylation of dihydroxyacetone phosphate by glyceronephosphate O-transferase (GNPAT) (Nagan and Zoeller, 2001). The ether is subsequently reduced to a vinyl-ether by an enzyme of the endoplasmic reticulum following export from the peroxisome (Gallego-García et al., 2019; Werner et al., 2020). Although diseases such as Parkinson’s and Alzheimer’s have several possible explanations for the associated plasmalogen deficiency, it has been suggested to result from impairment of the peroxisomal steps of the biosynthetic pathway (Kou et al., 2011; Dorninger et al., 2017a; Jo and Cho, 2019). Therefore, therapeutically providing an ether precursor bypasses the enzymes of the peroxisome, leaving the body to catalyze the reduction of the ether to the plasmalogen-defining vinyl-ether bond.

Despite extensive academic *in vitro* and *in vivo* studies examining multiple aspects of various plasmalogen extracts on both biochemical and behavioral endpoints, none have been scaled up and tested in a traditional safety program required for all new drug products. PPI-1011 is one of several fully synthetic plasmalogen precursors under development in our lab, which we have shown over the years to not only augment plasmalogens in multiple cell lines and species (Wood et al., 2011b; Wood et al., 2011c; Wood et al., 2011d; Wood et al., 2011e; Gregoire et al., 2015), but also exhibit a vast array of effects including neuroprotection (Miville-Godbout et al., 2016; Miville-Godbout et al., 2017; Nadeau et al., 2019), protection against inflammation and loss of dopaminergic neurons (Miville-Godbout et al., 2016; Miville-Godbout et al., 2017; Nadeau et al., 2019), shift towards non-amyloidogenic processing (Wood et al., 2011a), reduction of cholesterol (Mankidy et al., 2010), improvement of remyelination in a model of multiple sclerosis (Wood et al., 2011a), regulation of membrane protein function (Wood et al., 2011c), potent anti-dyskinetic effect when administered in combination with levodopa to primates treated with MPTP (Gregoire et al., 2015; Bourque et al., 2018) and normalization of behavior in plasmalogen-deficient mice (Fallatah et al., 2019). Furthermore, PPI-1011 has an acceptable safety profile and shows no toxicity or adverse effects to date, including doses up to 1,000 mg/kg in adult rats and 28-day repeat dosing at 400 mg/kg in primates (manuscript in preparation).

Although we have previously demonstrated brain uptake of PPI-1011 using a [^13^C]PPI-1011 as well as uptake to peripheral tissues (Wood et al., 2011b), there remains a general uncertainty about the *in vivo* pharmacokinetic behavior, absorption, and distribution of orally administered plasmalogens or ether precursors, as a quantitative assessment of pharmacokinetics has never been performed. Therefore, as part of our ongoing preclinical safety and toxicity program, and to better understand plasmalogen disposition and excretion after treatment, we report herein the pharmacokinetics and tissue distribution of [^14^C]PPI-1011 following both single and repeat oral dosing in Sprague-Dawley male rats.

## Materials and Methods

### Chemicals

PPI-1011 (1-*O*-hexadecyl-2-*O*-cis-4, 7, 10, 13, 16, 19-docosahexaenoyl-3-*O*-lipoyl glycerol) was synthesized at Laxai Life Sciences Pvt Ltd. using a previously described synthetic route (Wood et al., 2011a). Structure was confirmed by mass spectrometry and NMR analysis. [^14^C]PPI-1011 was synthesized by RTI International using [^14^C]sodium cyanide to introduce a single [^14^C] label onto the first carbon of palmitoyl alcohol, which was linked to the *sn*1 position of the glycerol backbone. [^14^C]PPI-1011 had a specific activity of 70.2 μCi/mg and a radiochemical purity of 95.9%. Handling of this material was in accordance with US Nuclear Regulatory Commission (NRC), Pennsylvania Bureau of Radiation Protection regulations, Frontage QWBA final study protocol, and all applicable Frontage Standard Operating Procedures (SOP).

Ethanolamine and 2-methoxyethanolamine were purchased from Sigma. Carbo-Sorb^®^ E, PermaFluor^®^ E+, Ultima Gold™, Hionic-Fluor, and Ultima-Flo™ M liquid scintillation cocktails were obtained from Perkin Elmer Life Sciences. Stopflow AQ liquid scintillation cocktails were purchased from AIM Research Company. Solvents used for chromatographic analysis were HPLC or ACS reagent grade and purchased from Fisher. All other reagents were of analytical or ACS reagent grade.

### Animals

All animal studies were performed by Frontage Laboratories using male Sprague-Dawley (SD) rats (Charles River Breeding Laboratories) 8–10 weeks old with body weights between 208 and 283 g. Animals were acclimated for a minimum of 3 days and examined to ensure their health status prior to dosing. The animal room was controlled to maintain a temperature of 20 ± 5°C, relative humidity between 30 and 70%, and a 12 h light/12 h dark cycle. Reverse osmosis filtered water and food (certified Purina Rodent Diet) were available to animals *ad libitum*. Final study plans were reviewed and approved by the Institutional Animal Care and Use Committee at Frontage Laboratories and complied with the applicable sections of the Final Rules of the Animal Welfare Act regulations (9 CFR) and were in accordance with the Association for the Assessment and Accreditation of Laboratory Animal Care recommendation.

### Dose Formulation

PPI-1011 and [^14^C]PPI-1011 were formulated together in liquid coconut oil (LCO) containing 0.1% 1-thioglycerol for oral dosing. For single dose studies, PPI-1011 and [^14^C]PPI-1011 were combined to generate an oral formulation with a concentration of 20 mg/ml and 40 μCi/ml on the day before dosing and stored at 4°C. Formulation for the repeat dosing QWBA experiment was made on the day prior to the first dose with a concentration of 20 mg/ml and 20 μCi/ml. This formulation was then aliquoted and stored frozen at −20°C until the day of use. Concentration and radiochemical purity of all formulations were confirmed by HPLC prior to dosing.

### Pharmacokinetic Study

The pharmacokinetics of [^14^C]PPI-1011 were performed in four rats that were surgically implanted with femoral artery catheters (FAC) for blood collection at least 2 days before dosing began. [^14^C]PPI-1011 was administered as a single oral dose at 100 mg/kg (200 μCi/kg). Blood samples were collected in EDTA tubes and centrifuged to obtain plasma at 1, 3, 6, 12, 18, 24, 48, and 72 h after drug administration. Duplicate aliquots of plasma (∼25 μl or higher) were transferred to liquid scintillation vials and the weights of sample aliquots were recorded. Aliquots were mixed with Ultima Gold (5 ml) as scintillation fluid and total radioactivity was determined by liquid scintillation counting (LSC) and used to calculate the total radioactive concentration.

### Mass Balance and Excretion Study

Four male rats were administered a single oral dose of [^14^C]PPI-1011 at 100 mg/kg (200 μCi/kg). Feces, urine, and the cage rinse samples were collected at pre-determined times up to 168 h post-dose (HPD). The expired air was collected using a carbon dioxide trapping apparatus equipped with airflow apparatus. Expired [^14^C]CO_2_ was collected for two of the four dosed animals for 24 h after the dose in a solution of ethanolamine:2-methoxyethanol (1:3, v/v) in two traps. Rats were sacrificed by overdose of CO_2_ and each cage was rinsed with 25% ethanol in water (>50 ml) at the end of the study. The cage rinse sample and the expired [^14^C]CO_2_ was stored at 4°C.

Diluted dose solutions, plasma, urine, and cage rinse were analyzed in duplicate by LSC, as described above. The expired CO_2_ was analyzed the same day it was collected by mixing a 1 ml aliquot of the carbon dioxide absorbents with 5 ml of Hionic-Fluor for radioactivity concentrations with LSC. Feces samples were homogenized in 5-fold volume of purified water then duplicate fecal homogenate aliquots were transferred to Combusto Cones and combusted with a Sample Oxidizer (Model A307, Perkin Elmer) equipped with an Oximate 80 Robotic Automatic Samples. The [^14^C]CO_2_ generated by the combustion was also collected in Carbo Sorb E and radioactivity was determined with PermaFluor as the scintillant using a Tri-Carb LSC.

### Single Dose Tissue Distribution Study

Six male rats were administered a single oral dose of [^14^C]PPI-1011 at 100 mg/kg (200 μCi/kg) and one rat was sacrificed at each of the following time points after the administration of the dose: 8, 12, 18, 24, 48, or 168 h. Terminal blood was collected by cardiac puncture directly into tubes with K_2_EDTA, an aliquot was stored at 4°C until LSC was performed for radioactivity, while the remaining blood was processed to plasma and stored at −20°C until LSC. Following blood collection, the carcasses were frozen in a cold bath with hexanes and dry ice for at least 8 min and then dried and stored at −70°C for at least 5 h. The carcasses were transferred to a −20°C freezer for at least 12 h before embedding.

### Repeat Dose Tissue Distribution Study

Three male rats were administered [^14^C]PPI-1011 at 100 mg/kg (100 μCi/kg) once daily for 15 days. One rat was sacrificed at each of the following time points after the administration of the day 15 dose: 24, 48, and 96 h. Terminal blood was collected by cardiac puncture directly into tubes with K_2_EDTA, an aliquot was stored at 4°C until LSC was performed for radioactivity, while the remaining blood was processed to plasma and stored at −20°C until LSC. Following blood collection, the carcasses were frozen in a cold bath with hexanes and dry ice for at least 8 min and then dried and stored at −70°C for at least 12 h. The carcasses were transferred to a −20°C freezer for at least 12 h before embedding.

### QWBA Sectioning and Quantification

QWBA was used to determine radioactivity concentrations in the caecum (contents and mucosa), large intestine (contents and mucosa), small intestine (contents and mucosa), stomach (contents and mucosa), brain, choroid plexus, spinal cord, bone, skin, adrenal gland, pineal body, pituitary gland, thyroid gland, liver, kidney (cortex, medulla and whole kidney), urinary bladder (contents and wall), brown fat, white fat, eye (lens, uveal tract and whole eye), epididymis, prostate gland, testis, ovary, uterus, lung, exorbital lachrymal gland, Harderian gland, pancreas, salivary gland, skeletal muscle, myocardium, cardiac blood, bone marrow, lymph node, spleen, and thymus. As well, LSC was used to determine the total radioactivity in the whole blood and plasma.

The whole-body cryosectioning technique described by (Ullberg, 1977) was the basis for embedding and cryosectioning the rats assigned to this study. Each frozen carcass was shaved, and then the limbs and tail were removed prior to embedding. Chilled (4°C) 3% CMC (low viscosity) was utilized to provide a firm support of reinforced ice around each rat and for a secure attachment to the microtome stage. Upon thorough freezing of the embedding medium surrounding each rat, the frozen CMC block was removed from the freezing bath and four 3/8 inch holes were drilled into the block. One cryosection quality control standard (CQCS) was prepared with a known amount of radioactivity and at least 1 ml aliquots were added to each of the four drilled holes. CQCS data were utilized to evaluate the overall performance of the QWBA assay.

Each frozen rat was sagittally trimmed (50–100 μm) at −20°C until exposure of a few target tissues employing a Leica Cryomacrocut heavy-duty microtome (Leica Microsystems Inc.,). Disposable stainless steel Feather type H45L knives (Feather Safety Razor Co., Ltd.) were utilized for trimming and sectioning each frozen rat. Several thinner sections (50 μm) were discarded prior to obtaining multiple 50 μm sections at various cryosectioning levels for QWBA analysis. Upon the collection of at least 60 target tissues in multiple cryosectioning levels, the cryosections were kept in the Cryomacrocut cryochamber for a minimum of 18 h for dehydration depending upon the number of cryosections within the cryochamber. After dehydration was complete, the cryosections were transferred to ambient temperature for processing. Mounted whole-body cryosections and a set of 15 standards were sandwiched between a standard phosphor screen and an exposure cassette. All cryosections were exposed to the phosphor screen for 4-days in a cabinet protected with lead prior to scanning using a Typhoon PhosphorImager (Cytiva). MCID™ Analysis software (InterFocus Ltd.) was utilized to generate a calibration curve of the STD nominal concentrations (nCi/g) versus PhosphorImager response (counts/μm^2^ minus background) by weighted (1/x) linear regression analysis (Potchoiba et al., 1995; Potchoiba et al., 1998). The linear regression curve was then utilized to determine the concentration (nCi/g) of total radioactivity in whole-body tissues and blood. Radioactivity concentrations were expressed as μg Eq/g based on the specific activity of the compound in the final dose formulations. Radioactivity concentrations represent the mean of all determinations for all whole-body cryosections that contained the target tissue or blood for each individual rat.

### Statistical Analysis

The PK calculations of total radioactivity in plasma were analyzed with non-compartmental analyses using Phoenix^®^ WinNonlin^®^ software (version 8.1). Statistical calculations for the other studies were limited to simple parameters such as means or averages, standard deviations (sd), and percentages (%). The amount of compound equivalents (Eq) in samples was calculated as concentrations expressed in μg Eq/g. Dose, MCID™ generated tissue concentrations, pharmacokinetic, and radioanalysis data were compiled using Microsoft Excel for Microsoft 365, Version 2010.

## Results

### Dose Formulation Analysis

Radiochemical concentrations and purity of the oral dose formulations were all confirmed to be >97% by HPLC with radioactivity detection. The mean radioactivity concentrations of dose formulations were 21.24 μCi/g. Representative radiochromatograms obtained from analysis of dose formulation of [^14^C]PPI-1011 are shown in Supplementary Figure S1. The retention time of [^14^C]PPI-1011 was approximately 12 min. The stability of the dose formulation at 20 mg/ml concentration was examined at 4°C and at −20°C for up to 24 h. The results are shown in Supplementary Figure S2. Under the experimental conditions utilized in this study, the radioactive purity of [^14^C]PPI-1011 at 20 mg/ml in LCO with 0.1% 1-thioglycerol dose vehicle was stable up to 24 h at 4°C (96.5%) and −20°C (97.2%). The actual dose administered to each rat in μCi as well as the animal weights are shown in Supplementary Table S1.

For the repeat dose tissue distribution study, the radiochemical purity of the pre-dose formulation on day one was 97.9%. After being stored at −20°C, the presence of [^14^C]PPI-1011 + O was detected in the pre-dose formulation on day seven and the post-dose formulations on day eight and on day 15 at levels ranging between 3.6 and 10.1%. The overall total radiochemical purity of these formulations ranged between 95.4 and 97.9%, confirming the stability of [^14^C]PPI-1011 over the initial 15 consecutive daily dose administration interval.

### Pharmacokinetic Profile

**FIGURE 1:**
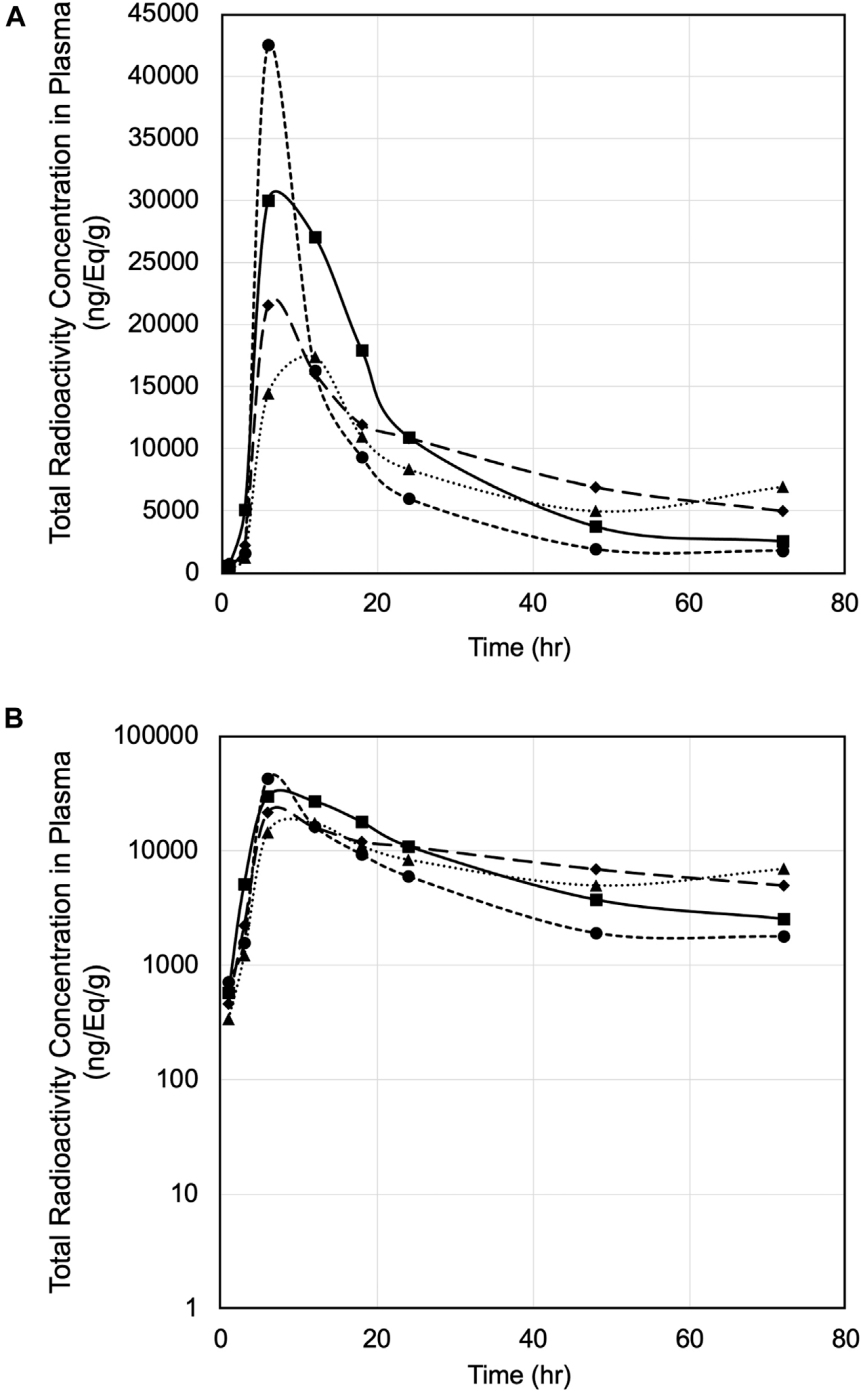
**(A)** Plasma concentration versus time of [^14^C]PPI-1011 in four male rats following single-dose oral administration at 100 mg/kg. **(B)** Log10 scale of A.

Pharmacokinetics of PPI-1011 were evaluated in male rats following a single oral dose of [^14^C]PPI-1011 at 100 mg/kg (200 μCi/kg). The total radioactivity concentrations in ng Eq/g in rat plasma up to 72 h are plotted in Figures 1A,B. The mean total radioactivity in plasma was approximately 500 ng Eq/g at 1 h, increased to ∼27,000 ng Eq/g at 6 h, and then decreased with time to ∼4,000 ng Eq/g at 72 h. Pharmacokinetics were characterized by a long elimination half-life (40 h), a mean t_max_ of 7.5 h, C_max_ of 27,900 ng/ml, and AUC_inf_ of 867,000 h*ng/ml (Table 1).

**TABLE 1:** Plasma pharmacokinetic parameters of total radioactivity following a single dose of 100 mg/kg [^14^C]PPI-1011 (200 μCi/kg) in male Sprague-Dawley rats.

	Rat 1	Rat 2	Rat 3	Rat 4	Mean	SD	%CV
AUC_last_ (h*ng/ml)	702,000	659,000	507,000	565,000	608,000	88,500	14.6
AUC_Inf_ (h*ng/ml)	753,000	953,000	542,000	1,220,000	867,000	289,000	33.3
AUC Extr (%)	6.8	31	6.5	54	20	20	92.5
MRT_Inf_ (h)	27	61	23	110	55	40	73
t_1/2_ (h)	18	42	19	80	40	29	73
t_max_ (h)	6.0	6.0	6.0	12	7.5	3.0	40
C_max_ (ng/ml)	30,000	21,500	42,500	17,400	27,900	88,500	39.8

### Recovery of Total Radioactivity

**FIGURE 2:**
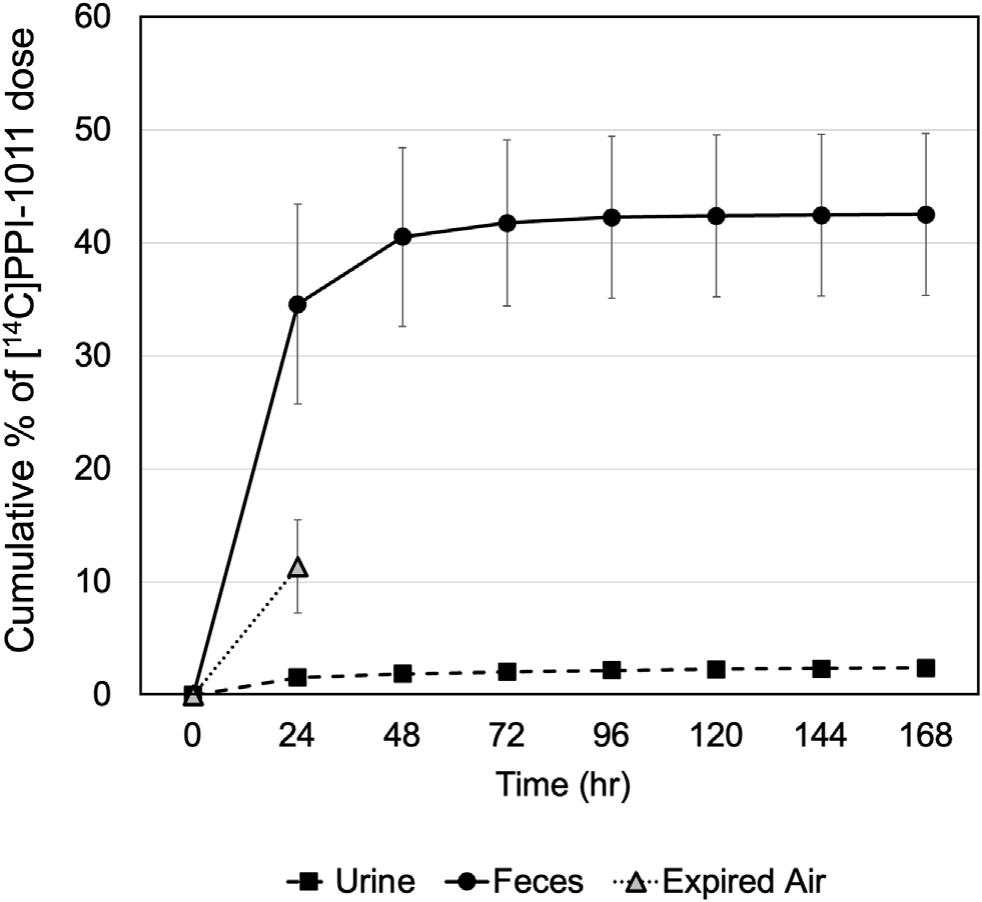
Recovery of radioactivity in excreta of male rats following a single oral dose of [^14^C]PPI-1011 at 100 mg/kg.

Recovery of total radioactivity was determined by measuring radioactivity excreted in the urine and feces as well as expired air (Figure 2). Following a single oral dose at 100 mg/kg (200 μCi/kg) of [^14^C]PPI-1011 in male SD rats, approximately 50% of dosed radioactivity (45.10 ± 7.34%) was recovered in urine and feces including cage rinses within the 168 h collection period. Approximately 11% of the dosed radioactivity was in expired air (as ^14^C-CO_2_) within 24 HPD, the only timepoint collected for expired air. Feces was the major excretion pathway and recovered 42.51 ± 7.16% of the dose, while a small amount of the dose (2.40 ± 0.59%) was found in urine up to day seven. Between 24 and 48 h, approximately 6% of the total dose was excreted in urine and feces, with little additional excretion observed between 48 and 168 h (<3%). A complete breakdown of the excretion data is available in Supplementary Table S2. The results indicate that significant amounts of dosed radioactivity were not recovered in male SD rats, suggesting high rate of absorption of [^14^C]PPI-1011 following oral administration, and that a large proportion of the absorbed material is retained within the tissue and serum beyond 7 days.

### Tissue Uptake and Distribution Following a Single Dose

**FIGURE 3:**
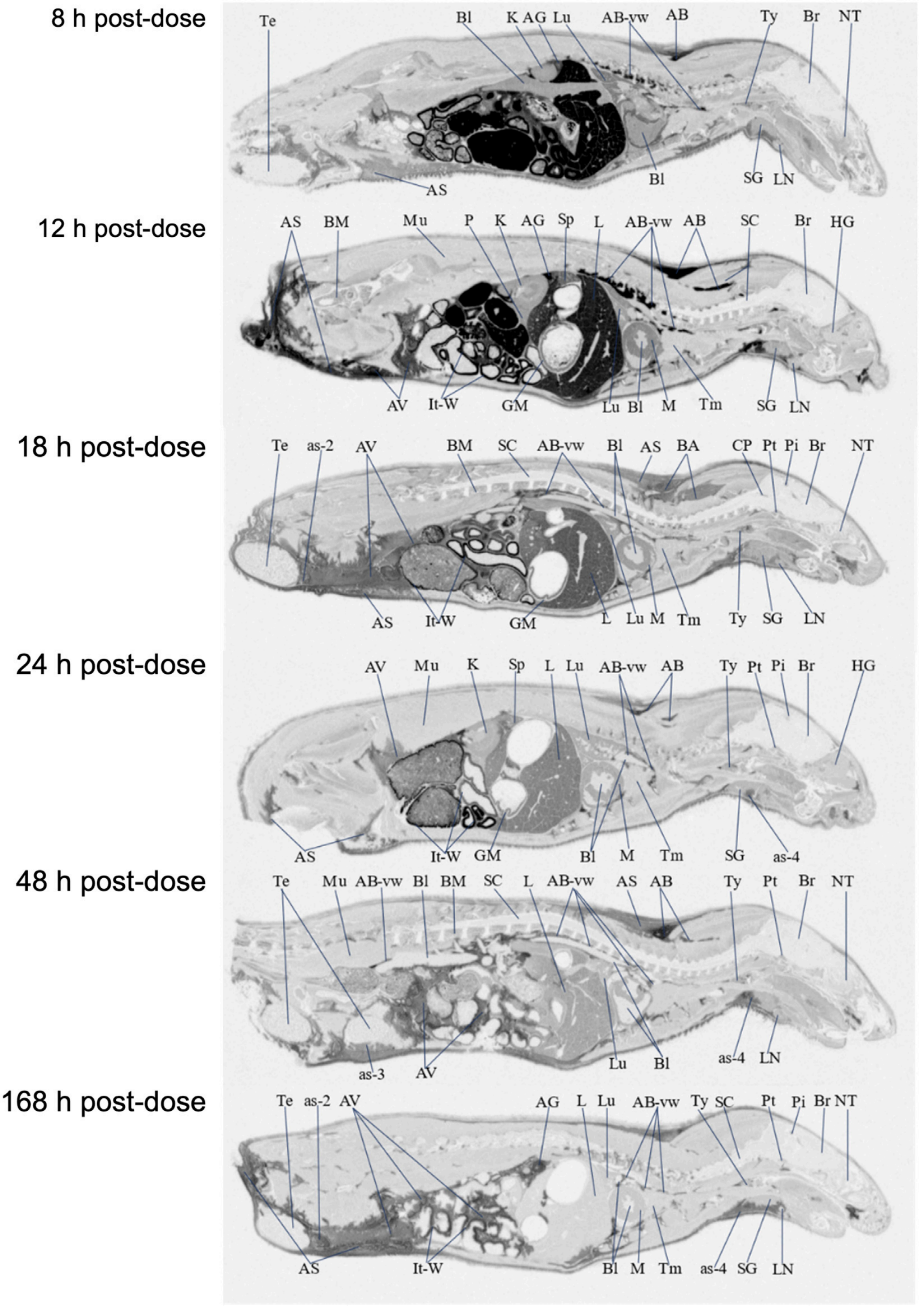
Selected autoradiography images (8, 12, 18, 24, 48, and 168 h post dose) of tissue distribution of in the whole rat body after single-dose oral administration at 100 mg/kg [^14^C]PPI-1011. The abbreviations are: AB, adipose brown; AS, adipose subcutaneous; AV, adipose visceral; AB-vw, adipose brown:vessel wall; as-2, adipose-soaked:epididymis; as-3, adipose-soaked:testis; as-4, adipose-soaked:salivary gland; AG, adrenal gland; Bl, blood; BM, bone marrow; Br, brain; CP, choroid plexus; GM, gastric mucosa; HG, harderian gland; It-W, intestinal tract wall; K, kidney; L, liver; LN, lymph node; Lu, lung; M, myocardium; Mu, muscle; NT, nasal tubinates; P, pancreas; Pi, pineal gland; Pt, pituitary gland; SC, spinal cord; SG, salivary gland; Sp, spleen; Te, testis; Tm thymus; Ty, thyroid.

[^14^C]PPI-1011 absorption across tissues was first determined by QWBA for up to 168 h following a single dose at 100 mg/kg. Compound-associated radioactivity was widely distributed into most evaluated tissues (*N* = 51) at the first sampling time of 8 HPD after oral administration of a single, bolus dose of [^14^C]PPI-1011 to Sprague-Dawley male rats (Table 2 and Figure 3). The highest concentrations of radioactivity occurred at 8 or 12 HPD in most tissues (*N* = 17 and 21, respectively) with some exceptions. Tissues with the highest peak concentrations of radioactivity were observed in gastrointestinal tract tissues, adipose (brown, subcutaneous, and visceral), adrenal gland, liver, and preputial gland. Tissues with the lowest peak concentrations of radioactivity were observed in the whole eye, the non-circumventricular CNS tissues, bone surfaces, testis, and uveal tract. Non-circumventricular CNS tissue was grouped separately from other CNS tissues due to differences in the blood-brain barrier. Rather than a true barrier, circumventricular CNS tissues are surrounded instead by fenestrated capillaries that result in their exposure to larger and more polar chemicals than other regions of the CNS (Benarroch, 2011). The ocular lens and vitreous body were the only tissues that were devoid of radioactivity throughout the course of this study. Although tissue radioactivity concentrations slowly declined over time in most tissues after reaching peak levels, compound-associated radioactivity was present in all but five tissues (bladder wall, bone surfaces, ocular lens, vitreous body, and seminal vesicles) at the last sampling time of 168 HPD.

**TABLE 2:** Radioactivity concentrations (μg equivalents/g) determined by quantitative whole-body autoradiography in tissues and blood through 168 h after a single oral dose of [^14^C]PPI-1011 to Group 1 Sprague-Dawley male rats (Nominal Dose Levels: 200 μCi/kg and 100 mg/kg).

Rat Identification:	01M	02M	03M	04M	05M	06M
Time (Post-Dose):	8 h	12 h	18 h	24 h	48 h	168 h
Adipose - Brown	171	426	66.0	133	132	89.0
Adipose - Brown (Vessel Wall)	301	383	94.2	110	98.6	76.7
Adipose - Visceral	83.1	170	75.6	71.6	111	90.7
Adipose - Subcutaneous	74.0	242	73.9	70.1	82.1	89.3
Adrenal Gland^a^	195	367	185	155	201	142
Bladder Wall	86.0	19.3	14.5	7.69	10.7	nd
Blood - QWBA	35.4	16.4	9.62	5.62	4.19	2.56
Bone Marrow	23.5	23.0	22.3	18.3	15.7	4.69
Bone Surface	2.30	1.15	1.25	0.513	0.508	nd
Diaphragm	55.6	59.9	40.4	37.4	23.0	11.7
Exorbital Lacrimal Gland	28.7	34.5	8.74	9.66	6.13	7.36
Harderian Gland	14.4	10.8	14.7	10.5	11.1	3.67
Intra-Orbital Lacrimal Gland	10.4	8.89	8.91	7.56	6.64	5.01
Kidney	42.9	45.3	36.1	24.7	24.6	14.5
Renal Cortex	41.4	46.6	38.1	26.8	24.3	15.3
Renal Medulla	51.0	53.2	23.0	25.8	22.7	11.8
Liver	313	269	121	73.4	40.0	7.50
Lung	74.0	51.9	45.4	30.2	22.9	11.2
Lymph Node	22.7	22.6	22.5	17.4	20.0	12.3
Muscle	10.3	12.6	9.63	7.57	8.42	7.01
Myocardium	92.0	72.6	42.4	31.0	26.6	13.4
Nasal Turbinates	16.0	10.6	6.43	4.81	5.03	4.29
Pancreas	66.3	52.7	39.8	24.8	28.0	18.2
Parotid Gland	39.1	30.4	35.2	20.5	20.9	12.3
Salivary Gland	53.3	31.4	28.8	21.3	22.9	15.4
Skin - Non-Pigmented	15.0	18.0	12.0	9.26	13.4	10.2
Spleen	105	111	71.3	46.3	33.1	9.36
Thymus	10.7	12.2	11.8	9.79	9.58	7.84
Thyroid	49.9	43.1	38.5	27.7	27.2	18.9
Ocular - Lens	nd	nd	nd	nd	nd	nd
Ocular - Uveal Tract	6.97	6.57	5.32	4.27	4.78	6.48
Ocular - Vitreous Body	nd	nd	nd	nd	nd	nd
Ocular - Whole Eye	1.83	2.03	1.65	1.14	1.30	1.55
Cerebellum	2.70	2.69	3.03	2.08	2.60	3.59
Cerebrum	2.39	2.22	2.49	1.61	2.23	2.94
Choroid Plexus	14.9	17.1	12.8	9.85	14.4	10.4
Colliculus	3.50	3.03	2.94	2.06	3.30	3.97
Medulla Oblongata	2.40	2.44	2.23	1.98	2.45	3.13
Olfactory Bulb	2.16	2.01	1.75	2.07	2.01	2.25
Pineal Gland	11.9	12.1	16.2	10.8	16.4	12.0
Pituitary Gland	21.2	21.2	22.0	16.7	19.2	12.9
Spinal Cord	2.71	2.76	3.20	1.75	2.56	3.33
Thalamus	2.27	2.31	2.20	1.71	1.91	2.78
Whole Brain	2.60	2.48	2.70	1.95	2.45	3.08
Epididymis	39.8	70.9	67.7	40.4	6.81	7.75
Preputial Gland	33.6	214	78.0	112	76.5	69.0
Prostate	49.6	11.8	28.0	56.7	7.15	4.44
Seminal Vesicles	12.8	2.99	as	5.38	9.12	nd
Testis	2.65	4.12	5.09	4.31	4.02	3.08
Cecum	as	450	177	231	34.3	as
Esophagus	17.4	16.9	15.0	9.46	10.8	5.34
Gastric Mucosa	91.2	67.8	70.1	48.7	37.5	14.5
Large Intestines	12.5	209	154	180	19.1	14.0
Small Intestines	609	347	239	247	43.3	139
Stomach	22.6	22.9	21.6	10.8	8.01	4.97

^a^as: tissue was soaked with adipose. nd: radioactivity was not detectable

Brown adipose, subcutaneous adipose, and visceral adipose had some of the highest concentrations of compound-associated radioactivity at all timepoints through the last sampling time of 168 HPD. The highest peak concentrations occurred at 12 HPD for all three adipose tissues. By 168 HPD, radioactivity declined approximately 5, 2, and 3 -fold in brown adipose, subcutaneous adipose, and visceral adipose, respectively, when compared with peak concentrations. Except for the adrenal gland, preputial gland, and small intestines, which had similar concentrations, the average last measured radioactivity concentrations in the three adipose tissues were 5 to 56 -fold greater than for all other tissues. This suggests that the retained radioactivity present in adipose tissue, along with its relatively slow release, may serve as a potential reservoir for continual re-distribution to other tissues prior to elimination.

The non-circumventricular CNS tissues (cerebellum, cerebrum, colliculus, medulla oblongata, olfactory bulb, spinal cord, and thalamus) had relatively low radioactivity concentrations throughout the study compared to other tissues. However, presence of radioactivity in CNS tissue at all samples times illustrated that [^14^C]PPI-1011 does distribute across the blood-brain barrier, and while levels in the non-circumventricular CNS tissue did not substantially change throughout the sampling times, they all demonstrated peak levels at 168 HPD. The choroid plexus, pineal gland, and pituitary gland are CNS tissues that reside behind leaky junctions of the blood-brain barrier. Concentrations of compound-associated radioactivity were higher (approximately 4-fold higher) in these three brain tissues than those measured in the non-circumventricular CNS tissues throughout the time course of this study. The highest peak concentrations occurred at 12, 18, and 48 HPD for the choroid plexus, pituitary gland, and pineal gland, respectively.

The ocular lens and vitreous body were devoid of radioactivity throughout the course of this study. Concentrations of compound-associated radioactivity present in the uveal tract did not substantially change between the first sampling time of 8 HPD through the last sampling time of 168 HPD. The distribution pattern for the whole eye was similar to the uveal tract, but radioactivity concentrations were 3 to 4 -fold lower than the uveal tract due to the lack of radioactivity present in other ocular tissues such as the lens and vitreous body.

Radioactivity concentrations were present in all male-specific tissues from the first sampling time of 8 HPD through the last sampling time of 168 HPD with one exception. By 168 HPD, the radioactivity concentration in the seminal vesicles declined below the lower limit of quantitation (LLOQ) of 0.485 μg Eq/g. The highest peak concentrations in the epididymis, preputial gland, testis, prostate, and seminal vesicles occurred at 12, 12, 18, 24, and 48 HPD, respectively. The testis was the only male-specific tissue where the concentrations of compound-associated radioactivity did not substantially change throughout the course of this study. These concentrations in the testis ranged from a low of 2.65 μg Eq/g at 8 HPD, to a high of 5.09 μg Eq/g at 18 HPD, with a C_last_ of 3.08 μg Eq/g at 168 HPD.

Radioactivity concentrations were present in the esophagus, gastrointestinal tract tissues, and the bladder wall from the first sampling time of 8 HPD through the last sampling time of 168 HPD with one exception. By 168 HPD, the radioactivity concentration in the bladder wall declined below the LLOQ of 0.485 μg Eq/g. The highest peak concentrations occurred in the bladder wall, esophagus, gastric mucosa, and small intestines at 8 HPD and in the cecum, large intestines, and stomach at 12 HPD. There was no detection of radioactivity in urine within the urinary bladder.

Concentrations of compound-associated radioactivity were present in blood from the first sampling time of 8 HPD through the last sampling time of 168 HPD. The highest blood concentration of compound-associated radioactivity occurred at 8 HPD with a concentration of 35.4 μg Eq/g. Blood concentrations of radioactivity declined at each subsequent sampling time with a last measurable concentration of 2.56 μg Eq/g at 168 HPD. Blood:plasma concentration ratios ranged from 0.685 to 1.11 from 8 to 48 HPD suggesting that there was not an apparent preferential uptake of [^14^C]PPI-1011 associated radioactivity into the cellular components of blood. By 168 HPD the blood:plasma concentration ratio more than doubled to 2.96 from the 48 HPD ratio suggesting an apparent uptake of [^14^C]PPI-1011 associated radioactivity into the cellular components of blood over time.

### Tissue Uptake and Distribution Following Repeat Dosing

**FIGURE 4:**
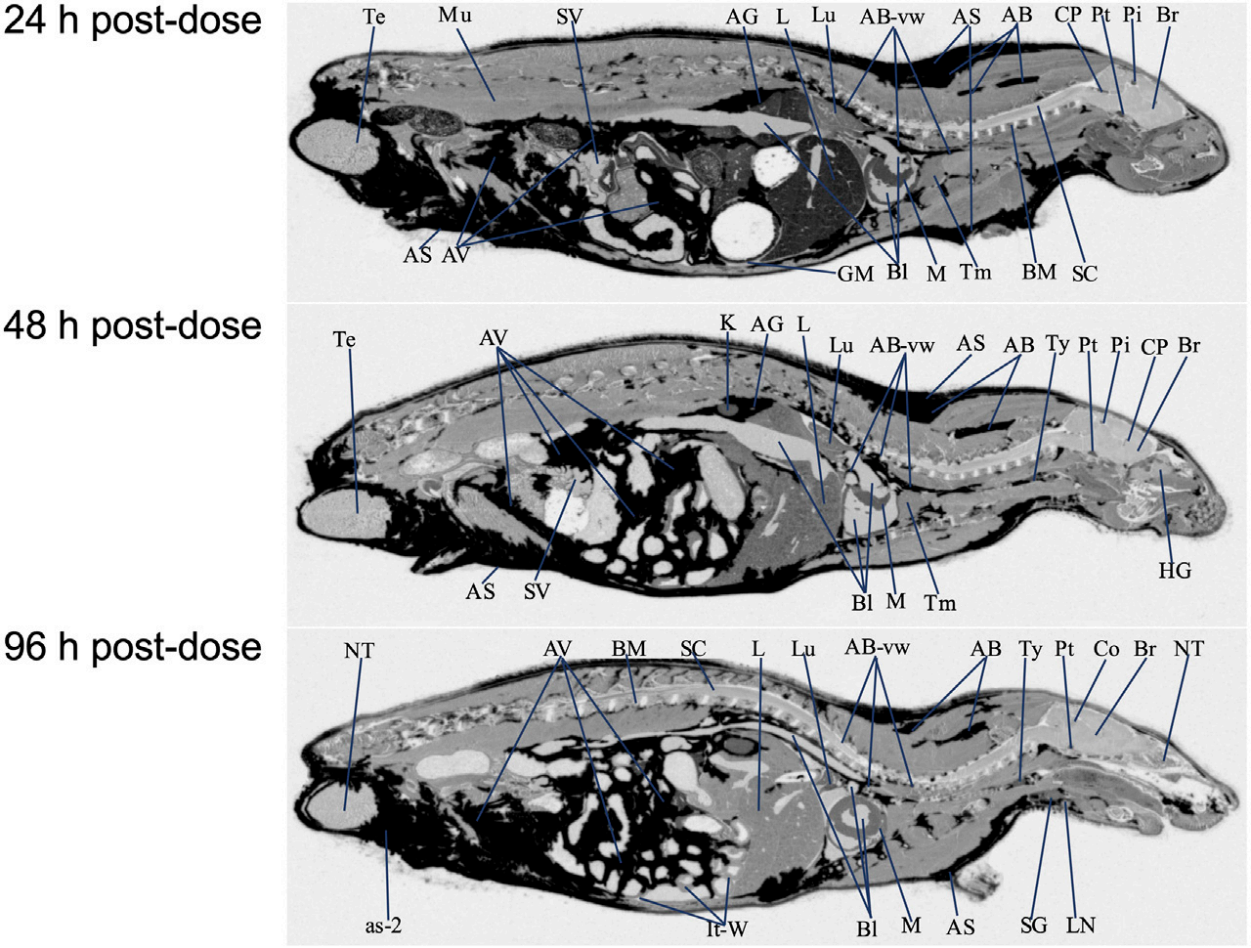
Selected autoradiography images (24, 48, and 96 h post final dose) of tissue distribution of in the whole rat body after 15 daily oral doses at 100 mg/kg [^14^C]PPI-1011. The abbreviations are: AB, adipose brown; AS, adipose subcutaneous; AV, adipose visceral; AB-vw, adipose brown:vessel wall; as-2, adipose-soaked:epididymis; AG, adrenal gland; Bl, blood; BM, bone marrow; Br, brain; Co, colliculus; CP, choroid plexus; GM, gastric mucosa; HG, harderian gland; It-W, intestinal tract wall; K, kidney; L, liver; LN, lymph node; Lu, lung; M, myocardium; Mu, muscle; NT, nasal tubinates; Pi, pineal gland; Pt, pituitary gland; SC, spinal cord; SG, salivary gland; SV, seminal vesicles; Te, testis; Tm thymus; Ty, thyroid.

QWBA was next used to determine [^14^C]PPI-1011 uptake following 15-consecutive daily oral bolus doses of [^14^C]PPI-1011 at 100 mg/kg (100 μCi/kg) to Sprague-Dawley male rats (Table 3 and Figure 4). Compound-associated radioactivity was present in all evaluated tissues (*N* = 54) at 24, 48, and 96 HPD. The highest concentrations of radioactivity occurred at 24 HPD in most tissues (*N* = 42) except for five tissues showing highest levels at 48 HPD (adrenal gland, bladder wall, nasal turbinates, subcutaneous adipose, and visceral adipose) and five tissues at 96 HPD (cerebrum, medulla oblongata, spinal cord, ocular lens, and prostate).

**TABLE 3:** Radioactivity concentrations (μg equivalents/g) determined by quantitative whole-body autoradiography in tissues and blood through 96 h after daily, multiple oral doses^1,2^ of [^14^C]PPI-1011 to Group 2 Sprague-Dawley male rats (Nominal Dose Levels: 100 μCi/kg and 100 mg/kg).

Rat Identification:	07M	08M	09M
Time (Post-Dose):	24 hr	48 hr	96 hr
Adipose - Brown	2211	1917	1301
Adipose - Brown (Vessel Wall)	1423	1017	1249
Adipose - Visceral	1402	1500	1265
Adipose - Subcutaneous	1316	1498	1373
Adrenal Gland^a^	1830	2346	1636
Bladder Wall	102	108	101
Blood - QWBA	55.0	34.3	29.5
Bone Marrow	160	113	77.3
Bone Surface	11.6	7.66	10.5
Diaphragm	204	172	125
Exorbital Lacrimal Gland	98.0	82.5	71.0
Harderian Gland	113	69.6	47.9
Intra-Orbital Lacrimal Gland	100	84.5	76.5
Kidney	304	221	238
Renal Cortex	323	244	250
Renal Medulla	238	183	186
Liver	412	186	129
Lung	217	181	191
Lymph Node	347	165	127
Muscle	115	95.4	97.9
Myocardium	292	222	172
Nasal Turbinates	180	184	80.2
Pancreas	355	312	333
Parotid Gland	296	257	220
Salivary Gland	308	271	227
Skin - Non-Pigmented	161	101	129
Spleen	319	209	147
Thymus	153	131	107
Thyroid	373	297	259
Ocular - Lens	5.82	5.05	6.92
Ocular - Uveal Tract	105	59.6	68.9
Ocular - Vitreous Body	2.55	2.01	2.06
Ocular - Whole Eye	22.7	15.8	18.2
Cerebellum	57.8	54.9	55.8
Cerebrum	45.7	39.9	47.5
Choroid Plexus	186	131	121
Colliculus	68.3	68.0	67.3
Medulla Oblongata	51.0	48.4	51.8
Olfactory Bulb	47.8	35.0	42.2
Pineal Gland	230	156	179
Pituitary Gland	266	226	191
Spinal Cord	46.8	51.2	53.0
Thalamus	45.4	42.3	45.4
Whole Brain	48.8	43.7	47.3
Epididymis	as	as	as
Preputial Gland	as	as	as
Prostate	74.6	72.1	84.8
Seminal Vesicles	63.2	60.7	54.7
Testis	70.3	47.2	43.3
Cecum	300	95.7	as
Esophagus	161	94.8	87.1
Gastric Mucosa	415	307	210
Large Intestines	255	122	94.3
Small Intestines	420	164	112
Stomach	133	78.9	109

^a^as: tissue was soaked with adipose.

Other than the adrenal gland, brown adipose, subcutaneous adipose, and visceral adipose had the highest concentrations of compound-associated radioactivity at 24, 48, and 96 HPD. The highest peak concentrations occurred at 24 HPD for brown adipose and at 48 HPD for subcutaneous and visceral adipose. By 96 HPD, radioactivity concentrations declined approximately 1.4, 1.1, and 1.2 -fold in brown adipose, subcutaneous adipose, and visceral adipose, respectively, when compared with peak concentrations. Except for the adrenal gland, the average last measured radioactivity concentrations in the three adipose tissues were 4 to 629 -fold greater than for all other tissues. Although tissue radioactivity concentrations slowly declined over time in most tissues after reaching peak levels, compound-associated radioactivity exhibited a greater than 2-fold decrease in only six tissues (bone marrow, Harderian gland, liver, lymph nodes, nasal turbinates, and spleen) at the last sampling time of 96 HPD. The data illustrated a retention of compound-associated radioactivity in all tissues after multiple doses at concentrations substantially greater than after a single oral dose.

Radioactivity present in the non-circumventricular CNS tissues did not substantially change in concentration across the three sampling times. This CNS concentration data showed that after multiple doses [^14^C]PPI-1011 radioactivity was present in greater concentrations, approximately 16 to 33 -fold higher at the 24 HPD timepoint, compared to concentrations at the same timepoint after a single oral dose.

After the 15-consecutive daily-dose administrations, radioactivity was now of measurable concentration in the ocular lens and vitreous body at the 24, 48, and 96 HPD. Concentrations of compound-associated radioactivity present in all ocular tissues did not substantially change in concentration across the three sampling times. The highest peak concentrations occurred at 24 HPD for the uveal tract, vitreous body, and whole eye and at 96 HPD for the ocular lens. The distribution pattern for the whole eye was similar to the uveal tract, but radioactivity concentrations were 4 to 5 -fold lower than the uveal tract due to the lower concentrations of radioactivity in other ocular tissues such as the lens and vitreous body.

Radioactivity concentrations were present in all the evaluated male-specific tissues at the three sampling times. However, due to adipose soaking, radioactivity concentrations were not measured in the epididymis and preputial gland at all sampling times. The highest peak concentrations occurred in the seminal vesicles and testis at 24 HPD and in the prostate at 96 HPD, respectively.

Radioactivity concentrations were present in esophagus, gastrointestinal tract tissues and the bladder wall at all three sampling times of 24, 48, and 96 HPD. The highest peak concentrations occurred in the cecum, esophagus, gastric mucosa, large intestines, small intestines, and stomach at 24 HPD and in the bladder wall at 48 HPD. There was no detection of radioactivity in urine within the urinary bladder.

Concentrations of compound-associated radioactivity were present in blood for all three sampling times of 24, 48, and 96 HPD. The highest blood concentration of compound-associated radioactivity occurred at 24 HPD with a concentration of 55.0 μg Eq/g. Blood concentrations of radioactivity declined at each subsequent sampling time with measurable concentrations of 34.3 μg Eq/g at 48 HPD and 29.5 μg Eq/g at 96 HPD. Blood:plasma concentration ratios were 2.31, 2.86, and 4.23 at 24, 48, and 96 HPD, respectively, suggesting that there was an apparent preferential uptake of [^14^C]PPI-1011 associated radioactivity into the cellular components of blood following multiple dose administration.

In general, compound-associated radioactivity was detectable in all tissues, including CNS, after 15-consecutive daily oral doses, and radioactivity concentrations were substantially greater when compared to those tissues from male rats after a single oral dose. This QWBA study illustrated that all tissues accumulated and retained [^14^C]PPI-1011 associated radioactivity after multiple doses.

## Discussion

We report herein the comprehensive pharmacokinetics and tissue distribution of synthetic plasmalogen precursor PPI-1011 following oral administration. Although previous research has addressed various aspects of the oral bioavailability, metabolism, and tissue incorporation of PPI-1011 *in vivo* (Wood et al., 2011b; Wood et al., 2011d; Gregoire et al., 2015; Miville-Godbout et al., 2016), the precise endogenous metabolic fate of PPI-1011 remained unknown. As far as we are aware, this is the first report of mass balance and tissue distribution using a radiolabeled orally-dosed plasmalogen precursor compound.

Pharmacokinetic analysis based on the single oral dose revealed key observations. First, the elimination half-life appears to be long, at approximately 40 h on average, but varied widely between 18 and 80 h. While there was a relatively high level of variation among the peak radioactivity levels between animals observed in the first 12 h, the overall PK profiles were similar. Furthermore, even at 72 h, radioactivity was still well above background. These observations match the previously reported time course of augmentation of the target 16:0/22:6 plasmalogen levels in rabbits following a single dose of PPI-1011, which peaked 12 h after a single dose and remained elevated at 48 h (Wood et al., 2011d).

The second key observation was that over half of the drug is retained and incorporated into the body following a single dose, matching historical estimates of uptake of other ether precursors (Das and Hajra, 1988). The vast majority of excretion of [^14^C]PPI-1011 was observed within the first 48 h after administration, with the primary route of excretion being in feces and only small amounts (<2.5%) detectable in urine. What did surprise us was the approximately 10% radioactivity present in expired air. Although we only had the 24-h timepoint, expired radioactivity past 24 h would likely have been minimal given the plateau in the total cumulative percent of dose excreted, and the relatively steady tissue levels observed between 24 and 168 h. Given the location of the [^14^C] label on the alpha carbon of the 16 carbon *sn*1 chain, the results suggest that the ether and/or vinyl-ether bond is cleaved, and the resulting fatty aldehyde/acid amenable to oxidation and release of radioactive CO_2_. We expand on this point later in the discussion. Overall, the rapid uptake, long half-life, and high absorption are all highly desirable PK characteristics for a therapy that is intended to systemically replenish deficient levels of an endogenous metabolite. While this [^14^C] data allowed for the first truly quantitative assessment of the PK characteristics of PPI-1011, it supports all of our previous work which has demonstrated that synthetic plasmalogens have good bioavailability (Wood et al., 2011d), tissue absorption (Wood et al., 2011a; Wood et al., 2011b; Fallatah et al., 2019), and plasmalogen augmentation (Fallatah et al., 2019; Miville-Godbout et al., 2017; Miville-Godbout et al., 2016; Gregoire et al., 2015, Wood et al., 2011e) *in vivo*. Further, we have previously demonstrated the pharmacodynamics of these compounds, which have been shown to result in behavioral (Gregoire et al., 2015; Bourque et al., 2018; Fallatah et al., 2019) and functional improvements within the cells (Wood et al., 2011a; Miville-Godbout et al., 2016; Miville-Godbout et al., 2017; Nadeau et al., 2019) of treated animals.

Although the elevation of plasmalogens in the circulation and tissues has been previously demonstrated following treatment with various supplements and precursors (including PPI-1011), the assessment of tissue distribution has been at best restricted to crude assays of homogenized whole tissues samples from a few target tissues (Brites et al., 2011; Fujino et al., 2017; Yamashita et al., 2017; Fujino et al., 2020; Todt et al., 2020; Nguma et al., 2021; Paul et al., 2021). This has prevented a true understanding of how orally administered ethers and plasmalogens are distributed *in vivo*. Our QWBA results represent the first visualization of plasmalogen uptake and metabolism *in vivo* over time. Following a single oral administration, uptake of PPI-1011 was observed widely throughout the body, with the ocular lens and vitreous body being the only two tissues without measurable levels at 8 h post-dose. Tissue levels peaked in most tissues between 8 and 12 h following a single administration of [^14^C]PPI-1011, mirroring the PK profile observed in plasma. Also, as observed in the PK study, significant radioactivity was still seen within tissues 7 days after a single dose, with only three tissues that had originally displayed [^14^C]PPI-1011 associated radioactivity (bladder wall, bone surfaces, and seminal vesicles) no longer showing detectable radioactivity.

Following 15 consecutive daily doses, the relative tissue distribution pattern mirrored that of the single dose, albeit with much higher uptake across all tissues as well as detectable radioactivity in the ocular lens and vitreous body. As expected by the lipophilic nature of plasmalogens, high levels of radioactivity were observed in adipose tissue, including brown adipose, subcutaneous adipose, and visceral adipose. Except for the adrenal gland, preputial gland, and small intestines, which had similar concentrations, the average last measured radioactivity concentrations in the three adipose tissues were 5 to 56 -fold greater than for all other tissues. This suggests that adipose tissue may act as a reservoir for plasmalogens or alkyl diacylglycerols, which are then slowly released over time and re-distributed to other tissues in the body as required. Interestingly, adipose tissue has been shown to be abnormal in severely plasmalogen deficient mice, with decreased size and shape of the adipocytes. Treatment with alkylglycerol was able to normalize these differences in adipocytes, which corresponded with a normalization of body weight in the animals (Brites et al., 2011), suggesting a physiological function for plasmalogens within adipose tissue. The rapid and robust uptake of PPI-1011 into adipose tissue could be clinically beneficial, particularly as a treatment for RCDP where patients have serious challenges maintaining their body weight (Duker et al., 2017).

Although comparing relative image intensity between the single dose and multiple dose cross sections must be approached with caution, it is worth noting the significant increase in retained radioactivity at 24 h and beyond following multiple doses compared to the single dose (compare Figure 3 to Figure 4 at 24 h). Although the 24-h timepoint following 15 doses did show the highest overall level of radioactivity in most tissues, the mean percentage radioactivity (normalized by tissue) at 96 h was still 75% that of the 24-h timepoint. This suggests that under a daily dosing regimen, tissues continue to take up and incorporate the drug throughout the body, and that due to the long half-life, withdrawal of compound up to 96 h appears to have little effect on overall levels in the body. One area worthy of future investigation would be studies aimed at better characterizing the ADME of multiple sequential doses to optimize dosing strategies for ensuring maximum augmentation at the lowest required dose.

Of particular interest in this study was the evaluation of whether the labeled ether backbone or resulting plasmalogens are capable of crossing the blood-brain barrier and incorporating into brain tissue. A historical ^13^C-labeled study suggested incorporation in a homogenized total brain sample (Wood et al., 2011b), but regional distribution in the brain of treated animals has never been possible due to analytical challenges. QWBA imaging allowed for a detailed evaluation of the distribution within the brain after single and repeated administration of [^14^C]PPI-1011. In both the single-dose and repeat-dose QWBA studies, there were detectable radioactivity concentrations present in these CNS tissues at all sampling times, confirming that the [^14^C]PPI-1011 ether backbone, and/or target plasmalogens, were able to cross the blood-brain barrier. Whole brain concentrations of total radioactivity were 4 to 6 -fold greater than the LLOQ of 0.485 μg Eq/g from 8 through 168 HPD, confirming uptake [^14^C]PPI-1011 to the brain after a single dose. Although this uptake to non-circumventricular CNS tissues (cerebellum, cerebrum, colliculus, medulla oblongata, olfactory bulb, spinal cord, and thalamus) was relatively low compared to other tissues, uptake following 15 doses in the brain was on average 16–18 times higher than after the single dose, further proving that metabolized PPI-1011 can make it to the brain in a dose-dependent manner. Previous work in a plasmalogen-deficient mouse model demonstrated that with chronic administration of an alkylglycerol over months, there was a robust augmentation of plasmalogen levels in peripheral tissue and a small but detectable increase in levels within the brain (Brites et al., 2011). These observations, combined with slow turnover and what appears to be the body’s ability to store reserves in adipose tissue, suggests that a daily dosing regimen over an extended period of time will result in plasmalogen augmentation in the CNS. Our work also suggests that minor changes in plasmalogen levels within the brain likely have a meaningful impact, with PPI-1011 demonstrating neuroprotective effects within the brain even when the difference in total plasmalogen levels did not reach statistical significance (Wood et al., 2011a; Miville-Godbout et al., 2016).

With respect to circumventricular tissues, the choroid plexus, pineal gland, and pituitary gland are CNS tissues that reside behind leaky junctions of the blood-brain barrier. Not surprisingly, concentrations of [^14^C]PPI-1011 associated radioactivity were substantially higher in these three brain regions than those measured in the non-circumventricular CNS tissues. Seven days following a single dose of [^14^C]PPI-1011, the average radioactivity concentration for choroid plexus, pineal gland, and pituitary gland was approximately 4-fold higher when compared with the average concentration for the non-circumventricular CNS tissues. This further supports that while the blood-brain barrier does partially impede the uptake of plasmalogen lipids, it does not prevent it.

Our study has a couple of limitations to address. First, the determination of various *in vivo* radiolabeled plasmalogen species originating from [^14^C]PPI-1011 was not possible within this study due to restrictions on processing radioactive samples. However, we have previously carried out numerous studies with PPI-1011 that have clearly demonstrated its ability to augment the entire *sn*1 16:0 plasmalogen pool (Gregoire et al., 2015; Miville-Godbout et al., 2016; Nadeau et al., 2019). In addition, we have previously evaluated the metabolic fate of the ether backbone *in vivo* and *in vitro* using a [^13^C] labeled version of PPI-1011 (Wood et al., 2011b). This study clearly demonstrated that the ether bond remains intact following oral administration and that once absorbed, remodeling of the glycerol backbone is limited to the *sn*2 position. Further, a more recent study using a [^13^C] labeled intact plasmalogen (PPI-1040) demonstrated that following absorption, rearrangement did not occur at the *sn*1 position (Fallatah et al., 2019). Additionally, loss of the *sn*3 phosphoethanolamine group was undetectable, suggesting that remodeling at the *sn*3 position is also minimal. However, we cannot exclude the possibility that the ether backbone is incorporated into non-ethanolamine glycerolipids. While it was outside of the scope of this study, we suggest the need for a future study using a [^13^C] labeled version of PPI-1011 to evaluate the metabolic fate of the ether backbone in non-phosphoethanolamine lipids.

A second limitation was our assumption that at least the majority of radioactivity uptake, as measured, represented intact plasmalogen. Previous studies with PPI-1011 have demonstrated a rapid reacylation of the *sn*2 fatty acid, but that removal of the *sn*1 was limited (Wood et al., 2011b; Wood et al., 2011d). Other reports using radiolabeled 1-*O*-alkyl-sn-glycerols have also concluded that absorption occurs without cleavage of the ether bond (Bergstrom and Blomstrand, 1956; Das et al., 1992). Nevertheless, our studies clearly demonstrate a large uptake to adipose tissue, of which it is reasonable to assume that the lipid species would predominantly be in an alkyl-acyl-acyl form given that adipose is primary a triglyceride storage depot (Dawkins and Stevens, 1966). This is consistent with studies by Paltauf who used tritium-labeled I-*O*-octadecyl *sn*-glycerol to conclude preferential acylation of the labelled precursor to form alkyl diacyl glycerols, whose stearic configuration is that of naturally occurring alkyl glycerol lipids (Paltauf, 1971). Our interpretation, therefore, based on our results herein and all historical data available to date, is that the absorbed radioactivity is represented by either alkyl diacyl species or by 1-alkenyl (vinyl-ether) containing phosphoglycerolipids. We also know that from previous PK studies in various species, PPI-1011 effectively results in detectable increases in circulating plasmalogen levels within hours of oral administration, confirming a rapid conversion to the *sn*1 vinyl-ether bond (Wood et al., 2011d; Gregoire et al., 2015). Our previous data using a [^13^C] radiolabeled vinyl-ether containing plasmalogen precursor, shows that the vinyl-ether is very stable endogenously, and that at least under normal physiological conditions, does not undergo rapid cleavage or rearrangement (Fallatah et al., 2019).

However, despite the historical evidence suggesting that the *sn*1 ether is maintained intact during absorption, we cannot disregard the expired radioactivity that we observed in this study. The only route by which this could occur would be through the fatty oxidation of the radiolabeled *sn*1 16:0 fatty acid. This would require cleavage of the ether, yielding an aldehyde that would likely undergo further oxidation to the acid by fatty aldehyde dehydrogenase (FALDH), followed by oxidation and subsequent release of the CO_2_ during the Krebb’s cycle. Alkylglycerol-mono-oxygenase (AGMO) has recently been reported to cleave alkylglyercols into a glyerol and fatty aldehyde (Tokuoka et al., 2013; Watschinger and Werner, 2013). AGMO is expressed primarily in the liver (Yu et al., 2019) making it likely that a small portion of the alkyl-acyl glycerol absorbed in the gastrointestinal tract following [^14^C]PPI-1011 administration was broken down in the liver by AGMO following first pass metabolism, leading to the observed radioactive CO_2_. Our PK results following a single oral dose showed that after 24 h, there was little further excretion in either the feces or urine. We would expect a similar observation for expired air, although the possibility that a small amount of radioactivity could have been expired post 24 h cannot be excluded. Our overall interpretation, therefore, is that relatively little of the compound-associated radioactivity would likely be due to the free 16:0 aldehyde following a cleavage event of the ether.

In summary, the results suggest that over half of the orally administered dose of PPI-1011 is retained by the body and that the ether backbone is well-absorbed in the gastrointestinal tract and redistributed throughout the body, including into CNS tissue. The long half-life (mean T_1/2_ of 40 h) along with high uptake to the adipose tissue suggests that the adipose tissue might be acting as a reservoir from which the body can continue to pull alkyl-diacyl glycerols for further incorporation into plasmalogens. Overall, this supports the use of PPI-1011 as an oral formulation for the augmentation of plasmalogens in deficient individuals, including those with neurodegenerative diseases that display deficient plasmalogen levels within the brain.

## Data Availability

The original contributions presented in the study are included in the article/Supplementary Material, further inquiries can be directed to the corresponding author.

## References

[B1] Benarroch E. E. (2011). Circumventricular Organs: Receptive and Homeostatic Functions and Clinical Implications. Neurology 77, 1198–1204. 10.1212/wnl.0b013e31822f04a0 21931109

[B2] Bergstrom S. Blomstrand R. (1956). The Intestinal Absorption and Metabolism of Chimyl Alcohol in the Rat. Acta Physiol. Scand. 38, 166–172. 10.1111/j.1748-1716.1957.tb01380.x 13394338

[B3] Bourque M. Grégoire L. DI Paolo T. (2018). The Plasmalogen Precursor Analog PPI-1011 Reduces the Development of L-DOPA-Induced Dyskinesias in De Novo MPTP Monkeys. Behav. Brain Res. 337, 183–185. 10.1016/j.bbr.2017.09.023 28917506

[B4] Bozelli J. C. Epand R. M. (2021). Plasmalogen Replacement Therapy. Membranes (Basel) 11. 10.3390/membranes11110838 PMC8620983 34832067

[B5] Braverman N. Steel G. Obie C. Moser A. Moser H. Gould S. J. (1997). Human PEX7 Encodes the Peroxisomal PTS2 Receptor and Is Responsible for Rhizomelic Chondrodysplasia Punctata. Nat. Genet. 15, 369–376. 10.1038/ng0497-369 9090381

[B6] Brites P. Ferreira A. S. Ferreira da Silva T. Sousa V. F. Malheiro A. R. Duran M. (2011). Alkyl-glycerol Rescues Plasmalogen Levels and Pathology of Ether-Phospholipid Deficient Mice. PLoS One 6, e28539. 10.1371/journal.pone.0028539 22163031 PMC3232224

[B7] Das A. K. Hajra A. K. (1988). High Incorporation of Dietary 1-O-Heptadecyl Glycerol into Tissue Plasmalogens of Young Rats. FEBS Lett. 227, 187–190. 10.1016/0014-5793(88)80895-0 3338573

[B8] Das A. K. Holmes R. D. Wilson G. N. Hajra A. K. (1992). Dietary Ether Lipid Incorporation into Tissue Plasmalogens of Humans and Rodents. Lipids 27, 401–405. 10.1007/bf02536379 1630273

[B9] Dawkins M. J. R. Stevens J. F. (1966). Fatty Acid Composition of Triglycerides from Adipose Tissue. Nature 209, 1145–1146. 10.1038/2091145a0 5925204

[B10] Dorninger F. Forss‐Petter S. Berger J. (2017a). From Peroxisomal Disorders to Common Neurodegenerative Diseases - the Role of Ether Phospholipids in the Nervous System. FEBS Lett. 591, 2761–2788. 10.1002/1873-3468.12788 28796901 PMC5856336

[B11] Dorninger F. Herbst R. Kravic B. Camurdanoglu B. Z. Macinkovic I. Zeitler G. (2017b). Reduced Muscle Strength in Ether Lipid-Deficient Mice Is Accompanied by Altered Development and Function of the Neuromuscular junction. J. Neurochem. 143, 569–583. 10.1111/jnc.14082 28555889 PMC5725694

[B12] Dragonas C. Bertsch T. Sieber C. C. Brosche T. (2009). Plasmalogens as a Marker of Elevated Systemic Oxidative Stress in Parkinson's Disease. Clin. Chem. Lab. Med. 47, 894–897. 10.1515/CCLM.2009.205 19575554

[B13] Duker A. L. Niiler T. Eldridge G. Brereton N. H. Braverman N. E. Bober M. B. (2017). Growth Charts for Individuals with Rhizomelic Chondrodysplasia Punctata. Am. J. Med. Genet. 173, 108–113. 10.1002/ajmg.a.37961 27616591

[B14] Fabelo N. Martín V. Santpere G. Marín R. Torrent L. Ferrer I. (2011). Severe Alterations in Lipid Composition of Frontal Cortex Lipid Rafts from Parkinson's Disease and Incidental Parkinson's Disease. Mol. Med. 17, 1107–1118. 10.2119/molmed.2011.00119 21717034 PMC3188884

[B15] Fallatah W. Smith T. Cui W. Jayasinghe D. DI Pietro E. Ritchie S. A. (2019). Oral Administration of a Synthetic Vinyl-Ether Plasmalogen Normalizes Open Field Activity in a Mouse Model of Rhizomelic Chondrodysplasia Punctata. Dis. Model. Mech. 13, dmm042499. 10.1242/dmm.042499 PMC6994958 31862688

[B16] Fujino T. Hossain M. S. Mawatari S. (2020). “Therapeutic Efficacy of Plasmalogens for Alzheimer’s Disease, Mild Cognitive Impairment, and Parkinson’s Disease in Conjunction with a New Hypothesis for the Etiology of Alzheimer’s Disease,” in Peroxisome Biology: Experimental Models, Peroxisomal Disorders and Neurological Diseases. Editor LIZARD G. (Cham: Springer International Publishing). 10.1007/978-3-030-60204-8_14 33417216

[B17] Fujino T. Yamada T. Asada T. Tsuboi Y. Wakana C. Mawatari S. (2017). Efficacy and Blood Plasmalogen Changes by Oral Administration of Plasmalogen in Patients with Mild Alzheimer's Disease and Mild Cognitive Impairment: A Multicenter, Randomized, Double-Blind, Placebo-Controlled Trial. EBioMedicine 17, 199–205. 10.1016/j.ebiom.2017.02.012 28259590 PMC5360580

[B18] Gallego-García A. Monera-Girona A. J. Pajares-Martínez E. Bastida-Martínez E. Pérez-Castaño R. Iniesta A. A. (2019). A Bacterial Light Response Reveals an Orphan Desaturase for Human Plasmalogen Synthesis. Science 366, 128–132. 31604315 10.1126/science.aay1436

[B19] Glaser P. E. Gross R. W. (1994). Plasmenylethanolamine Facilitates Rapid Membrane Fusion: a Stopped-Flow Kinetic Investigation Correlating the Propensity of a Major Plasma Membrane Constituent to Adopt an HII Phase with its Ability to Promote Membrane Fusion. Biochemistry 33, 5805–5812. 10.1021/bi00185a019 8180209

[B20] Glaser P. E. Gross R. W. (1995). Rapid Plasmenylethanolamine-Selective Fusion of Membrane Bilayers Catalyzed by an Isoform of Glyceraldehyde-3-Phosphate Dehydrogenase: Discrimination between Glycolytic and Fusogenic Roles of Individual Isoforms. Biochemistry 34, 12193–12203. 10.1021/bi00038a013 7547960

[B21] Goodenowe D. B. Cook L. L. Liu J. Lu Y. Jayasinghe D. A. Ahiahonu P. W. K. (2007). Peripheral Ethanolamine Plasmalogen Deficiency: a Logical Causative Factor in Alzheimer's Disease and Dementia. J. Lipid Res. 48, 2485–2498. 10.1194/jlr.p700023-jlr200 17664527

[B22] Grégoire L. Smith T. Senanayake V. Mochizuki A. Miville-Godbout E. Goodenowe D. (2015). Plasmalogen Precursor Analog Treatment Reduces Levodopa-Induced Dyskinesias in Parkinsonian Monkeys. Behav. Brain Res. 286, 328–337. 10.1016/j.bbr.2015.03.012 25771209

[B23] Han X. (2005). Lipid Alterations in the Earliest Clinically Recognizable Stage of Alzheimers Disease: Implication of the Role of Lipids in the Pathogenesis of Alzheimers Disease. Car 2, 65–77. 10.2174/1567205052772786 15977990

[B24] Hermetter A. Rainer B. Ivessa E. Kalb E. Loidl J. Roscher A. (1989). Influence of Plasmalogen Deficiency on Membrane Fluidity of Human Skin Fibroblasts: a Fluorescence Anisotropy Study. Biochim. Biophys. Acta (Bba) - Biomembranes 978, 151–157. 10.1016/0005-2736(89)90510-5 2914126

[B25] Jo D. S. Cho D.-H. (2019). Peroxisomal Dysfunction in Neurodegenerative Diseases. Arch. Pharm. Res. 42, 393–406. 10.1007/s12272-019-01131-2 30739266

[B26] Kou J. Kovacs G. G. Höftberger R. Kulik W. Brodde A. Forss-Petter S. (2011). Peroxisomal Alterations in Alzheimer's Disease. Acta Neuropathol. 122, 271–283. 10.1007/s00401-011-0836-9 21594711 PMC3168371

[B27] Lohner K. Balgavy P. Hermetter A. Paltauf F. Laggner P. (1991). Stabilization of Non-bilayer Structures by the Etherlipid Ethanolamine Plasmalogen. Biochim. Biophys. Acta (Bba) - Biomembranes 1061, 132–140. 10.1016/0005-2736(91)90277-f 1998688

[B28] Lohner K. Hermetter A. Paltauf F. (1984). Phase Behavior of Ethanolamine Plasmalogen. Chem. Phys. Lipids 34, 163–170. 10.1016/0009-3084(84)90041-0

[B29] Mankidy R. Ahiahonu P. W. Ma H. Jayasinghe D. Ritchie S. A. Khan M. A. (2010). Membrane Plasmalogen Composition and Cellular Cholesterol Regulation: a Structure Activity Study. Lipids Health Dis. 9, 62. 10.1186/1476-511x-9-62 20546600 PMC2902472

[B30] Miville-Godbout E. Bourque M. Morissette M. AL-Sweidi S. Smith T. Jayasinghe D. (2017). Plasmalogen Precursor Mitigates Striatal Dopamine Loss in MPTP Mice. Brain Res. 1674, 70–76. 10.1016/j.brainres.2017.08.020 28830769

[B31] Miville-Godbout E. Bourque M. Morissette M. AL-Sweidi S. Smith T. Mochizuki A. (2016). Plasmalogen Augmentation Reverses Striatal Dopamine Loss in MPTP Mice. PLoS One 11, e0151020. 10.1371/journal.pone.0151020 26959819 PMC4784967

[B32] Nadeau J. Smith T. Lamontagne-Proulx J. Bourque M. AL Sweidi S. Jayasinghe D. (2019). Neuroprotection and Immunomodulation in the Gut of Parkinsonian Mice with a Plasmalogen Precursor. Brain Res. 1725, 146460. 10.1016/j.brainres.2019.146460 31525350

[B33] Nagan N. Zoeller R. A. (2001). Plasmalogens: Biosynthesis and Functions. Prog. Lipid Res. 40, 199–229. 10.1016/s0163-7827(01)00003-0 11275267

[B34] Nguma E. Yamashita S. Han K.-H. Otoki Y. Yamamoto A. Nakagawa K. (2021). Dietary Ethanolamine Plasmalogen Alleviates DSS-Induced Colitis by Enhancing Colon Mucosa Integrity, Antioxidative Stress, and Anti-inflammatory Responses via Increased Ethanolamine Plasmalogen Molecular Species: Protective Role of Vinyl Ether Linkages. J. Agric. Food Chem. 69, 13034–13044. 10.1021/acs.jafc.1c04420 34723501

[B35] Paltauf F. (1971). Metabolism of the Enantiomeric 1-O-Alkyl Glycerol Ethers in the Rat Intestinal Mucosa *In Vivo*; Incorporation into 1-O-Alkyl and 1-O-Alk-1′-Enyl Glycerol Lipids. Biochim. Biophys. Acta (Bba) - Lipids Lipid Metab. 239, 38–46. 10.1016/0005-2760(71)90190-1 5569939

[B36] Paul S. Rasmiena A. A. Huynh K. Smith A. A. T. Mellett N. A. Jandeleit-Dahm K. (2021). Oral Supplementation of an Alkylglycerol Mix Comprising Different Alkyl Chains Effectively Modulates Multiple Endogenous Plasmalogen Species in Mice. Metabolites 11. 10.3390/metabo11050299 PMC8148155 34066368

[B37] Potchoiba M. J. Tensfeldt T. G. Nocerini M. R. Silber B. M. (1995). A Novel Quantitative Method for Determining the Biodistribution of Radiolabeled Xenobiotics Using Whole-Body Cryosectioning and Autoradioluminography. J. Pharmacol. Exp. Ther. 272, 953–962. 7853213

[B38] Potchoiba M. J. West M. Nocerini M. R. (1998). Quantitative Comparison of Autoradioluminographic and Radiometric Tissue Distribution Studies Using Carbon-14 Labeled Xenobiotics. Drug Metab. Dispos 26, 272–277. 9492392

[B39] Rog T. Koivuniemi A. (2016). The Biophysical Properties of Ethanolamine Plasmalogens Revealed by Atomistic Molecular Dynamics Simulations. Biochim. Biophys. Acta (Bba) - Biomembranes 1858, 97–103. 10.1016/j.bbamem.2015.10.023 26522077 PMC4673105

[B40] Todt H. Dorninger F. Rothauer P. J. Fischer C. M. Schranz M. Bruegger B. (2020). Oral Batyl Alcohol Supplementation Rescues Decreased Cardiac Conduction in Ether Phospholipid‐deficient Mice. Jrnl Inher Metab. Disea 43, 1046–1055. 10.1002/jimd.12264 PMC7540404 32441337

[B41] Tokuoka S. M. Kita Y. Shindou H. Shimizu T. (2013). Alkylglycerol Monooxygenase as a Potential Modulator for PAF Synthesis in Macrophages. Biochem. Biophysical Res. Commun. 436, 306–312. 10.1016/j.bbrc.2013.05.099 23743196

[B42] Ullberg S. (1977). The Technique of Whole Body Autoradiography. Cryosectioning Large Specimen. Sci. Tools, LKB Instrument J., 2–28.

[B43] Wanders R. J. A. Dekker C. Hovarth V. A. P. Schutgens R. B. H. Tager J. M. VAN Laer P. (1994). Human Alkyldihydroxyacetonephosphate Synthase Deficiency: a New Peroxisomal Disorder. J. Inherit. Metab. Dis. 17, 315–318. 10.1007/bf00711817 7807941

[B44] Wanders R. J. A. Schumacher H. Heikoop J. Schutgens R. B. H. Tager J. M. (1992). Human Dihydroxyacetonephosphate Acyltransferase Deficiency: a New Peroxisomal Disorder. J. Inherit. Metab. Dis. 15, 389–391. 10.1007/bf02435984 1405476

[B45] Watschinger K. Werner E. R. (2013). Alkylglycerol Monooxygenase. IUBMB Life 65, 366–372. 10.1002/iub.1143 23441072 PMC3617469

[B46] Werner E. R. Keller M. A. Sailer S. Lackner K. Koch J. Hermann M. (2020). TheTMEM189gene Encodes Plasmanylethanolamine Desaturase Which Introduces the Characteristic Vinyl Ether Double Bond into Plasmalogens. Proc. Natl. Acad. Sci. USA 117, 7792–7798. 10.1073/pnas.1917461117 32209662 PMC7149458

[B48] Wood P. L. Khan A. M. Mankidy R. Smith T. Goodenowe D. (2011a). “Plasmalogen Deficit: A New and Testable Hypothesis for the Etiology of Alzheimer's Disease,” in Alzheimer's Disease Pathogenesis-Core Concepts, Shifting Paradigms and Therapeutic Targets. Editor DE LA MONTE S. (InTech).

[B49] Wood P. L. Khan M. A. Smith T. Ehrmantraut G. Jin W. Cui W. (2011b). *In Vitro* and *In Vivo* Plasmalogen Replacement Evaluations in Rhizomelic Chrondrodysplasia Punctata and Pelizaeus-Merzbacher Disease Using PPI-1011, an Ether Lipid Plasmalogen Precursor. Lipids Health Dis. 10, 182. 10.1186/1476-511x-10-182 22008564 PMC3238230

[B50] Wood P. L. Khan M. Smith T. Goodenowe D. B. (2011c). Cellular Diamine Levels in Cancer Chemoprevention: Modulation by Ibuprofen and Membrane Plasmalogens. Lipids Health Dis. 10, 214. 10.1186/1476-511x-10-214 22087745 PMC3231815

[B51] Wood P. L. Smith T. Lane N. Khan M. A. Ehrmantraut G. Goodenowe D. B. (2011d). Oral Bioavailability of the Ether Lipid Plasmalogen Precursor, PPI-1011, in the Rabbit: a New Therapeutic Strategy for Alzheimer's Disease. Lipids Health Dis. 10, 227. 10.1186/1476-511x-10-227 22142382 PMC3260122

[B52] Wood P. L. Smith T. Pelzer L. Goodenowe D. B. (2011e). Targeted Metabolomic Analyses of Cellular Models of Pelizaeus-Merzbacher Disease Reveal Plasmalogen and Myo-Inositol Solute Carrier Dysfunction. Lipids Health Dis. 10, 102. 10.1186/1476-511x-10-102 21682894 PMC3141545

[B53] Yamashita S. Hashimoto M. Haque A. M. Nakagawa K. Kinoshita M. Shido O. (2017). Oral Administration of Ethanolamine Glycerophospholipid Containing a High Level of Plasmalogen Improves Memory Impairment in Amyloid β-Infused Rats. Lipids 52, 575–585. 10.1007/s11745-017-4260-3 28551706

[B54] Yu H. Dilbaz S. Coßmann J. Hoang A. C. Diedrich V. Herwig A. (2019). Breast Milk Alkylglycerols Sustain Beige Adipocytes through Adipose Tissue Macrophages. J. Clin. Invest. 129, 2485–2499. 10.1172/jci125646 31081799 PMC6546455

