# Supplementary figures and images for: Pharmacokinetics, Mass Balance, Excretion, and Tissue Distribution of Plasmalogen Precursor PPI-1011
Source: Front Cell Dev Biol. 2022 Apr 25;10:867138. doi: 10.3389/fcell.2022.867138 (PMC9081329; doi:10.3389/fcell.2022.867138)

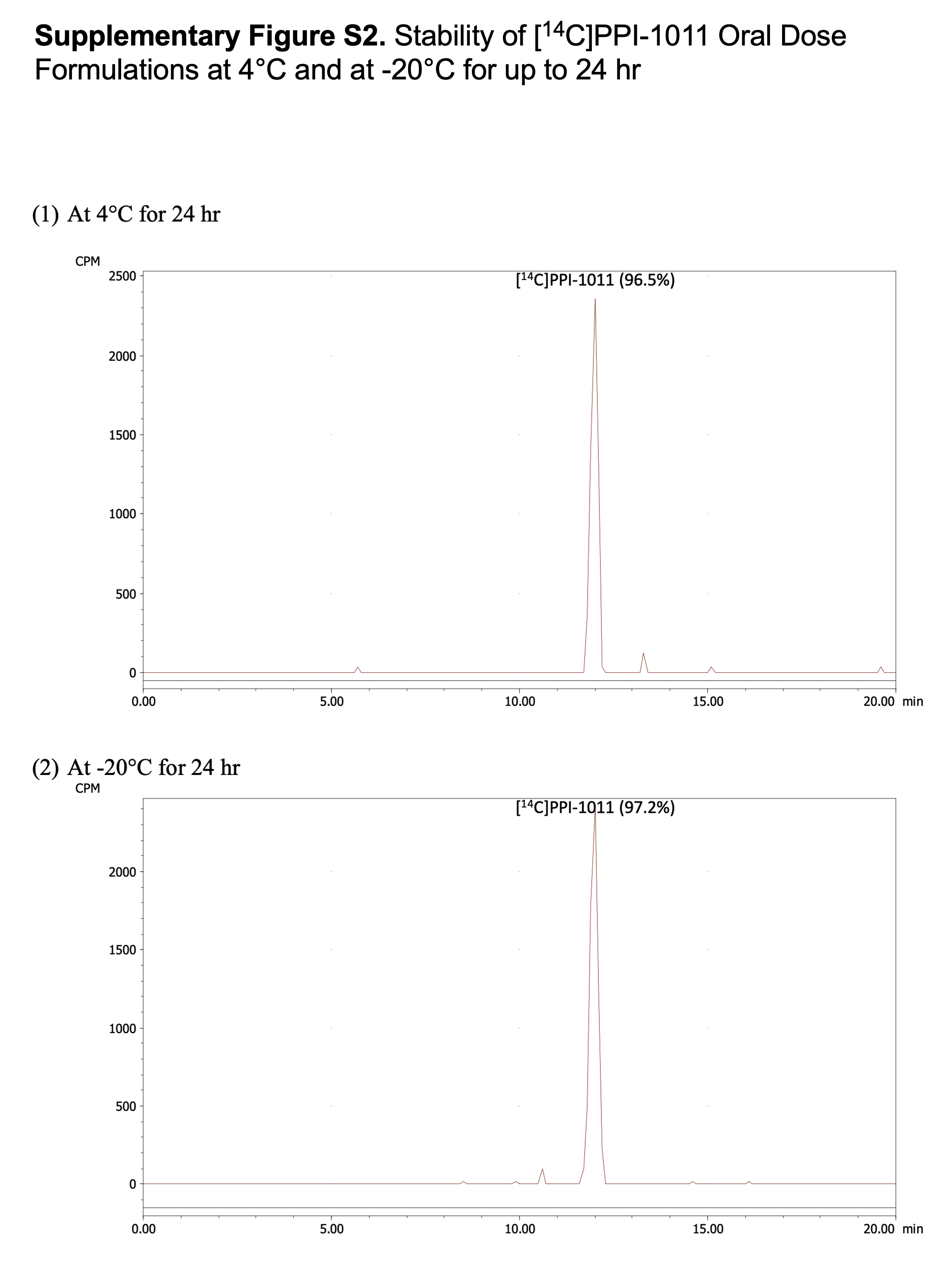

Supplement: Supplementary file 1 [file Image2.jpg]

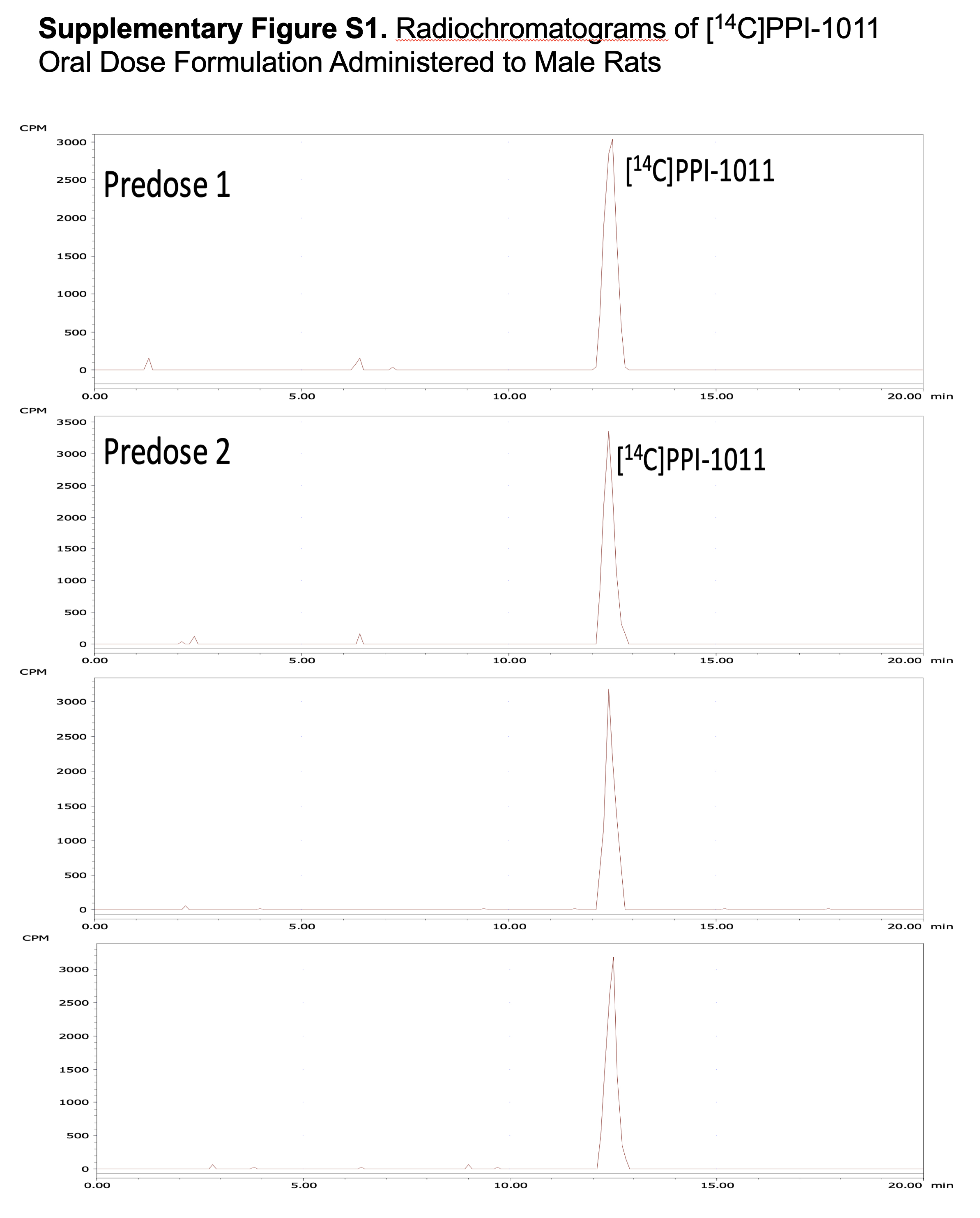

Supplement: Supplementary file 4 [file Image1.jpg]
