# Supplementary material for: Pharmacokinetics, Mass Balance, Excretion, and Tissue Distribution of Plasmalogen Precursor PPI-1011
Source: Front Cell Dev Biol. 2022 Apr 25;10:867138. doi: 10.3389/fcell.2022.867138 (PMC9081329; doi:10.3389/fcell.2022.867138)
Supplement: Supplementary file 2 [file Table1.pdf]

Supplementary Table S1. Rat IDs, weights and doses administered to rats.

Group #	Frontage Rat #	Rat Weight	Target Dose Vol	Syringe + Dose Weight	Empty Syringe Weight	Actual Dose Weight per Rat	Dose Conc.	µCi/Rat Dosed	µCi/kg Rat Weight	% Diff from Target Radioactive Dose*
		kg	mL	g	g	g	µCi/g	µCi	µCi/kg	%
G1	1M	0.208	1.0	4.9316	3.9813	0.9503	42.62	40.50	195	-2.64
	2M	0.274	1.4	5.2726	3.9697	1.3029		55.53	203	1.33
	3M	0.259	1.3	5.1391	3.9058	1.2333		52.56	203	1.48
	4M	0.280	1.4	5.3626	3.9904	1.3722		58.48	209	4.44
G2	5M	0.264	1.3	5.1362	3.9142	1.2220		52.08	197	-1.36
	6M	0.263	1.3	5.1790	3.9576	1.2214		52.06	198	-1.03
	7M	0.236	1.2	5.0734	3.9463	1.1271		48.04	204	1.78
	8M	0.266	1.3	5.2233	3.9774	1.2459		53.10	200	-0.18

*The target dose was 200 µCi/kg.
