# Supplementary material for: Pharmacokinetics, Mass Balance, Excretion, and Tissue Distribution of Plasmalogen Precursor PPI-1011
Source: Front Cell Dev Biol. 2022 Apr 25;10:867138. doi: 10.3389/fcell.2022.867138 (PMC9081329; doi:10.3389/fcell.2022.867138)
Supplement: Supplementary file 3 [file Table2.pdf]

Supplementary Table S2. Recovery of radioactivity (cumulative percent of dose) in excreta of male rates following a single 100 mg/kg oral dose of [^{14}C]PPI-1011 (Group 2).

Group 2: % of Dose							
Matrix	Time (hr)	5M	6M	7M	8M	**Mean**	SD
Urine	0	0.00	0.00	0.00	0.00	**0.00**	0.00
	0-8	0.69	0.86	0.34	0.05	**0.48**	0.36
	0-24	2.04	1.73	1.33	1.09	**1.55**	0.42
	0-48	2.37	2.13	1.67	1.34	**1.88**	0.46
	0-72	2.59	2.36	1.82	1.49	**2.06**	0.50
	0-96	2.72	2.51	1.91	1.57	**2.18**	0.53
	0-120	2.80	2.60	1.98	1.63	**2.26**	0.54
	0-144	2.90	2.74	2.04	1.68	**2.34**	0.58
	0-168	2.96	2.82	2.08	1.73	**2.40**	0.59
Feces	0	0.01	0.00	0.00	0.00	**0.00**	0.00
	0-24	47.26	27.14	33.48	30.45	**34.58**	8.84
	0-48	49.70	30.99	43.24	38.18	**40.53**	7.91
	0-72	50.31	33.17	44.52	39.04	**41.76**	7.35
	0-96	50.50	33.92	45.09	39.48	**42.25**	7.14
	0-120	50.63	34.05	45.22	39.60	**42.37**	7.14
	0-144	50.69	34.13	45.30	39.69	**42.45**	7.14
	0-168	50.79	34.18	45.34	39.72	**42.51**	7.16
CO2	0	0.00	0.00			**0.00**	0.00
	0-24	8.47	14.28			**11.37**	4.10
Cage Rinse	168	0.35	0.19	0.09	0.16	**0.20**	0.11
Total	0	0.01	0.00	0.00	0.00	**0.00**	0.00
	0-24	49.30	28.87	34.81	31.54	**36.13**	9.11
	0-48	52.08	33.12	44.90	39.53	**42.41**	8.05
	0-72	52.90	35.52	46.33	40.53	**43.82**	7.49
	0-96	53.22	36.43	47.00	41.05	**44.42**	7.29
	0-120	53.43	36.66	47.20	41.23	**44.63**	7.28
	0-144	53.60	36.87	47.33	41.37	**44.79**	7.27
	0-168*	54.10	37.19	47.51	41.61	**45.10**	7.34
	0-168**	62.57	51.47			**57.02**	7.85

*Total at 0-168 hr included urine, feces and cage rinse.
** Total at 0-168 hr included urine, feces, cage rinse, and expired air (^{14}C-CO_2 for Rat #5M and 6M only; expired air (^{14}C-CO_2) were not collected for Rat #7M and 8M.
